# The effect of interaural timing on the posterior auricular muscle reflex in normal adult volunteers

**DOI:** 10.1371/journal.pone.0194965

**Published:** 2018-04-04

**Authors:** T. P. Doubell, A. Alsetrawi, D. A. S. Bastawrous, M. A. S. Bastawrous, A. Daibes, A. Jadalla, J. W. H. Schnupp

**Affiliations:** 1 School of Medicine, Royal College of Surgeons in Ireland-Medical University of Bahrain, Busaiteen, Kingdom of Bahrain; 2 Department of Biomedical Science, City University of Hong Kong, Kowloon, Hong Kong; 3 Department of Physiology, Anatomy and Genetics, University of Oxford, Oxford, United kingdom; Johns Hopkins University, UNITED STATES

## Abstract

The posterior auricular muscle (PAM) reflex to sounds has been used clinically to determine hearing threshold as an alternative to other audiological diagnostic measures such as the auditory brainstem response. We have shown that the PAM response is also sensitive to interaural timing differences in normally hearing adults. PAM responses were evoked by both ipsilateral/ contralateral monaural stimulation and by binaural stimulation. Introducing sound delays ipsilaterally or contralaterally decreased the PAM response amplitude and increased its latency. The PAM response in this study shows a qualitatively similar pattern to that seen by the binaural interaction component (BIC) of the auditory brainstem potential to binaural clicks described in previous studies, in that both: have their shortest latency and maximal amplitudes centred around zero interaural timing differences, have response latencies increase with increasing interaural delays up to 1.2 ms and have response amplitudes decrease with increasing interaural delays of up to 1.2 ms. Our data show that the PAM response may be useful in measuring binaural integration in humans non-invasively for diagnostic or research studies.

## Introduction

Binaural hearing uses interaural timing (ITD) and intensity differences (ILD) that occur between the two ears to localise sounds in the horizontal plane [1]. Sound location is also necessary for spatial streaming, in which different sound sources relative to their spatial locations can be further separated and identified. Streaming allows the person to focus their attention on one particular target sound amongst a cacophony of distracter sounds [2]. Fundamental to this process is the binaural integration of signals from the left and right ears which is thought to occur in the auditory brainstem nuclei [3,4].

Binaural hearing tasks are often affected in disorders of central auditory processing [5]. Brainstem binaural processing can be measured physiologically using the binaural interaction component of the auditory brainstem response (ABR)[6]. The binaural interaction component (BIC) of the ABR can be computed by subtracting the monaural sums of the left and right ear evoked ABRs from the binaural evoked ABR [7–11]. This produces a multi-peaked binaural difference waveform with a response approximately in the position of waves IV and V of the human ABR [10].

The BIC is thought to result from electrical activity of a population of brainstem binaural neurons integrating ITD and ILDs, processing signal underlying roles in binaural fusion and sound lateralisation [9,12]. The exact anatomical location of the BIC generating neurons is unknown but the mostly commonly studied component (DN1) of the BIC occurs during wave IV of the ABR in animal models and wave V in humans, which is thought to be generated by the superior olivary nucleus (SON) and nucleus of the lateral lemniscus (NLL) [13]. The click evoked BIC amplitude and latency vary with both ITD and ILD [10,12] Some studies suggest the BIC has a perceptual correlate, only being present for ITDs that allow binaural fusion as they lie within a physiological range of ITDs (maximal ITD ≈ 0.8 ms). Authors reported measurable BIC of ITDs up to 0.8 ms [12], and even 1.0 ms [14,15]. However, using chirp stimuli and taking care to optimize the signal to noise ratio of the ABR recording, the BIC is measurable beyond the approximate physiological maximum ITD of 0.8 ms, up to 1.5 ms in humans [16]. Similarly, animal studies have shown measurable click evoked BIC beyond the physiological maximal ITDs in cats [17] and guinea pigs [18].

The BIC of the ABR is so far the only direct physiological measure of binaural interaction that can be measured non-invasively in humans in laboratory and clinical settings with relatively inexpensive equipment. Because of this, the BIC has been used in to study normally hearing adults [10,11], normally hearing neonates [19,20] and clinical populations [21–24]. The signal to noise ratio of the BIC is poor, making it difficult to measure, nevertheless the BIC is the main physiological diagnostic measure available for binaural processing in humans [6,25] and animals [26]. The poor signal to noise ratio of the BIC can be improved somewhat with increased signal averaging and objective threshold criteria [6,11,15] and chirp stimuli [16,27], but measuring it accurately nevertheless remains challenging. Another potential confound arises from the fact that the main measurable peak in the BIC may decrease in amplitude with increasing ITD [6,25], making the BIC more variable and harder to detect as ITD increase. Measuring BICs at numerous ITDs would involve a lengthy recording procedure which may be impracticable in many clinical situations. Due to these difficulties, BIC measurements are not routinely performed clinically [28].

Several sound-evoked muscle reflexes have traditionally also been used to probe the functionality of the auditory system including the startle reflex, middle ear reflex and the pinna reflex. Middle ear reflex pathways route auditory signals through the cochlear nucleus and superior olivary complex before synapsing onto facial and trigeminal motor neurons. The pathway for the pinna reflex is less well-known, particularly in humans, but auricular motor neurons in the facial nucleus probably receive input from multiple auditory nuclei (cochlear nucleus, superior olivary complex and inferior colliculus), and may include the reticular formation and the superior colliculi [29–33].

In this study we investigate an alternative approach to the non-invasive study of binaural integration by recording the sensitivity of an auditory reflex; the sound-evoked posterior muscle (PAM) response [34–39] to binaural stimulus parameters. The PAM response is thought to be part of a vestigial pinna reflex in humans, and in this study we document the effect of changing ITD on the click-evoked PAM reflex.

## Methods and materials

### Subjects

Adult volunteers were recruited via advertisements at the Royal College of Surgeons in Ireland-Medical University of Bahrain (RCSI-MUB). Initially 25 healthy adults with no previous history of hearing loss or neurological disorder were recruited. Participants read an information sheet and signed an informed consent form at the beginning of the laboratory sessions. Ethics approval was granted for this study by the research ethics committee of the RCSI-MUB in accordance with the standards laid down in the Declaration of Helsinki. Volunteers undertook an audiogram (Amplivox 116 audiometer, Eynsham, UK). Subjects were included in the study if they had normal pure tone audiogram thresholds, averaged RMS baseline noise < 10 μV and in further testing had significant posterior auricular muscle electromyography responses to all the 9 experimental conditions. Some subjects were not included because the PAM response had a small amplitude, requiring a greater amount of signal averaging. After exclusion, 11 participants (4 females and 7 male) remained whose data were analysed.

### Recording apparatus and EMG

Before electrode placement the skin was prepared with abrasive scrub (Nuprep, Weaver and company, Colorado, USA), washed and dried. Three surface electrodes (Ag-AgCL 15x20 mm disposable discs, Spes Medica, Battipaglia, Italy) were attached; (1) the active electrode approximately over the PAM, (2) the reference electrode on the posterior surface of the pinna and (3) an earth electrode on the neck, using the method described by O’Beirne and Patuzzi, 1999. Briefly the PAM electrode was placed approximately 1 cm posterior to the junction of the pinna and scalp, just level with the roof of the external meatus. The reference electrode was placed on the posterior surface of the pinna level with the PAM electrode, in order to target the tendinous insertion of the PAM where the EMG may reverse in sign [36]. In this experiment we only recorded from the left PAM and, during the recordings, subjects sat in a chair with the back of their head supported in a relaxed position. In additional subjects were asked to deviate their eyes to gaze to the left side for the duration of the experiment as in the method of O’Beirne and Patuzzi, 1999.

Electrode impedances were kept below 3 kΩ, and voltages were recorded with a preamplifier (Medusa PA4-LI, Tucker-Davis-Technologies (TDT), Alachua, USA) and digitised with a real time processor (RZ6, TDT, Alachua, USA) at a sampling rate to 25 KHz and an overall gain of 100,000. Raw voltage epochs were acquired using neurophysiology software (Brain ware, TDT, Alachua, USA) before offline filtering (low pass 500Hz and high pass 10 HZ). No normalisation or further smoothing was done. Subjects were tested with their eyes deviated to the side to increase PAM response amplitude. If subjects with an average RMS noise greater than 10 μV were not included in the analysis. Additionally, some subjects were not included in the analysis because their responses were not significantly different from baseline after averaging 500 repeat trials. To limit the duration of experiment we limited the number of repeat trials to 500 for the nine stimuli conditions, although with more repeats it might have been possible to gain significant responses in more subjects. We generally found the PAM response to be greatest with the subject sitting as opposed to lying and in an alert state rather the being sleepy. On two occasions our subjects fell asleep and the PAM amplitude was greatly reduced. These conditions made it difficult to reduce interference from neighbouring neck and facial muscles as is normally done when recording the auditory brainstem response.

### Stimulus generation

Stimuli were generated digitally using a real time processor (TDT RZ6, TDT, Alachua, USA) and Rpvds software (Rpvds, TDT, Alachua, USA). Stimulus files were then run in the data acquisition software (Brainware, TDT, Alachua, USA) to record PAM electromyographic responses. Stimuli used were 0.1 ms clicks (alternating positive and negative polarity rectangular pulses) at a sampling rate of 25 KHz. Binaural clicks were produced, and time delays introduced to the left or right channels to generate interaural timing differences. Nine different interaural timing differences (ITD’s) were used (-1.6ms, -1.2 ms, -0.8 ms, -0.4 ms, 0 ms, 0.4 ms, 0.8 ms, 1.2 ms, 1.6 ms) with minus denoting sounds presented earlier to the ipsilateral side of the recording electrode. During a recording session, each of the 9 ITD conditions were presented 500 times in a random interleaved single block which would last for 15–20 minutes. The stimulus presentation rate was 5 Hz. Click sounds were presented through insert ear phones (Eartone 3A, 3M, Bracknell, UK). Sound pressure level was measured with a sound pressure meter and 2cc coupler (Bruel & Kjaer Sound & Vibration, Naerum, Denmark). Clicks were presented at 40 dB nHL to avoid crosstalk between the ears.

Each stimulus was presented 500 times to produce a grand average of 5500 samples per ITD condition from the group of 11 subjects. All offline data processing, graphics, analysis and statistics were done in custom written programs (Matlab, The Mathworks Inc., Matick, USA).

### Statistical analysis

The PAM response most often shows an initial negative peak followed by a positive peak. We show the data as recorded and do not invert the sign as is sometimes the practice. From each sweep we measured the following variables; the peak to peak amplitude, minimum amplitude (1st peak amplitude), and maximum amplitude (2^nd^ peak amplitude), minimum latency (1^st^ peak latency), maximum latency (2^nd^ peak latency) during discrete response periods. The response periods were as follows; 14–17 ms for minimum amplitude and latency, 19–22 for maximum amplitude and latency and 14–22 ms for peak to peak amplitude. Statistical analysis involved performing within subjects (repeated measures) ANOVA with either ITD or binaural/monaural stimulation condition as the factor. If ANOVAs were significant for the ITD or binaural/monaural stimulation condition further *post hoc* tests were performed using the Tukey’s honesty significant test for multiple comparisons. Data shown are means with the 95% confidence intervals as error bars.

## Results

### General observation of the PAM response

**Fig 1:**
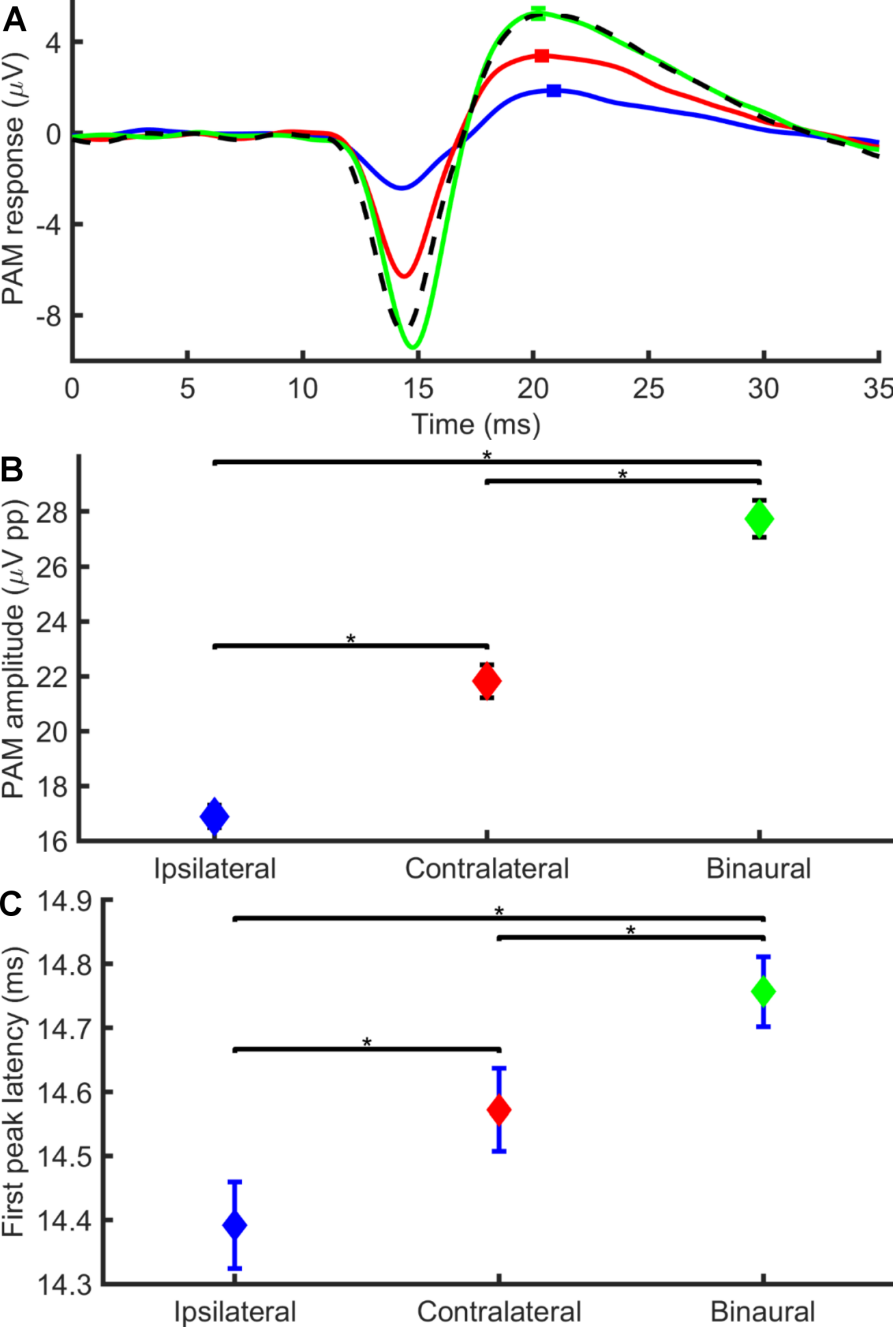
Average PAM response waveforms for monaural and binaural conditions. In 1A the averaged click evoked response to ipsilateral (blue line) and contralateral (red line) monaural stimulation and to binaural (green line) stimulation (n = 11 participants) are shown. When recorded with the positive electrode over the muscle and the negative electrode on the posterior pinna surface the response is biphasic with an initial negative deflection followed by a positive deflection. The linear summation of the ipsilateral (I) and contralateral(C) responses is shown (black dotted line). In 1B the average peak to peak amplitude of the click evoked PAM response are shown (n = 11 participants). On average binaural stimulation (green diamond) gave the greatest response followed by contralateral (red diamond) and then ipsilateral stimulation (blue diamond). In 1C the average latency of the first peak of the click evoked PAM response are shown. On average binaural stimulation (green diamond) gave the slowest response followed by contralateral (red diamond) and then ipsilateral stimulation (blue diamond). Error bars show the 95% confidence interval. Horizontal bars indicate pairwise Tukey’s comparisons and asterisks indicate significance at p<0.05.

We obtained a typically bi-phasic response in most subjects (negative-positive), although some individuals exhibited additional longer latency peaks. Our electrode montage had the positive electrode over the PAM and the negative electrode on the posterior surface of the ear, and we did not invert the waveform. For comparison with other studies we also low pass filtered our data (at 500 Hz) which has been shown to attenuate the full PAM response (Beirne and Patuzzi 1999) but leave the main components. We focussed our analysis on these main components; the first negative peak which had an approximate latency of 15 ms and a second positive peak with an approximate latency of 20 ms (see Fig 1A) and its resultant peak to peak amplitude. In the terminology of Thornton our first peak is equivalent to the N1 and our second peak is the P2 (Thornton 1975).

### Comparison of amplitude, and first and second peak latencies between binaural, ipsilateral and contralateral stimulation

Ipsilateral stimulation on average gave a smaller peak to peak response than contralateral at a similar latency for the negative and positive peaks (see Fig 1A). Linear summation of the ipsilateral and contralateral waveforms gave a predicted binaural response which was smaller and earlier in time by 0.4 ms than the actual binaural response. On average binaural stimulation produced a greater peak to peak amplitude response (M = 2.75 x 10^−5^ V, SD = 2.55 x 10^−5^) than either the ipsilateral (M = 1.70 x 10^-5^V, SD = 1.52 x 10^−5^) and contralateral (M = 2.16 x 10^−5^ V, SD = 2.23 x 10^−5^) stimulation (see Fig 1B). This effect of stimulation was significant with an ANOVA (F _(10, 2)_ = 837.27, p<0.0001). *Post hoc* tests showed the binaural, ipsilateral and contralateral response amplitudes to be significantly different from each other (p<0.05).

The first negative peak latency for the binaural stimuli (M = 14.79 ms, SD = 2.72) was also longer than with monaural ipsilateral (M = 14.30 ms, SD = 3.38) or contralateral (M = 14.48 ms, SD = 3.12) stimulation (Fig 1C). The effect of binaural/monaural stimulation on first peak latency was significantly different (F _(10, 2)_ = 38.24, p<0.0001). *Post hoc* tests showed the binaural, ipsilateral and contralateral latencies to be significantly different from each other (p<0.05). No significant effects of binaural/monaural stimulation were seen in the second positive peak latencies with ANOVA (F _(10, 2)_ = 0.1, p = 0.91).

### Effect of ITD on the binaural evoked PAMR

#### Peak to peak amplitude

**Fig 2:**
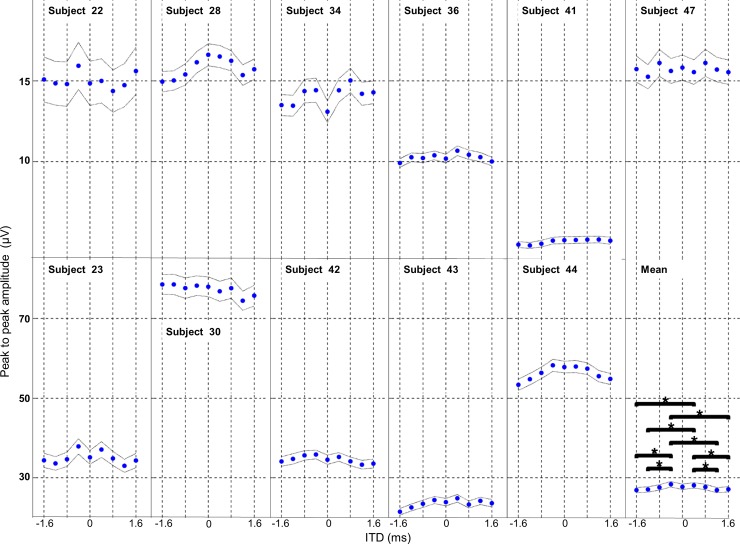
Effect of ITD on average peak to peak amplitude. The average peak to peak amplitude of the PAM response is shown for 9 ITD conditions for each of the 11 experimental subjects and the overall group mean. Negative ITD’s indicate an ipsilateral delay (sound in perceived as coming from the contralateral side to the recorded PAM). Positive ITDs indicate a contralateral delay (sound in perceived as coming from the ipsilateral side to the recorded PAM). Horizontal bars indicate pairwise Tukey’s comparisons and asterisks indicate significance at p<0.05. Fine dotted lines show the 95% confidence intervals.

We tested 9 ITD conditions, including ipsilateral ear and contralateral ear leading conditions, on the PAM response (see Fig 2) in 11 subjects. Data for individual subjects with a smaller peak to peak amplitude PAM response are shown in the top row of Fig 2, whereas subjects with larger peak to peak amplitude PAM responses are shown on the bottom row of Fig 2. The overall averaged PAM responses from all subjects is shown in the lower right corner (Mean). The mean response (subject averaged data) shows the peak to peak amplitude was greatest at 0.4 and -0.4 ms ITDs and became smaller at greater ipsilateral or contralateral delays. The effect of the ITD condition on the group average PAM peak to peak amplitude was significant with an ANOVA (F _(10, 8)_ = 7.73, p<0.001). For summary statistics see supporting information S1A Table. *Post hoc* Tukey tests revealed significant differences between 0.4ms ITD and -1.6, -1.2, and 1.2 ms ITD conditions and between -0.4 ms ITD and -1.6, -1.2, 1.2 and 1.6 ms ITD conditions (p<0.05) for the group data (see Fig 2 panel labelled Mean). The 0, 0.8, -0.8 ms ITD condition showed no significant differences with other ITD conditions for the group data. There was no significant difference between 0 ITD condition and +/-0.4 ms ITDs for the group data. Summary data and Tukey p values for pairwise comparison of ITD conditions are shown in the supporting information S1A Table. The individual data showed the peak to peak amplitudes of the PAM response varied between the 11 subjects. However, the group data trend for higher amplitude peak to peak responses around -0.4, 0, and +0.4 ITD’s and smaller responses at ITDs greater than _+/- 0.4 ms longer was seen in some of the subjects (Subjects 23, 28, 34, 36, 43 and 43 in Fig 2).

#### First and second peak amplitudes

**Fig 3:**
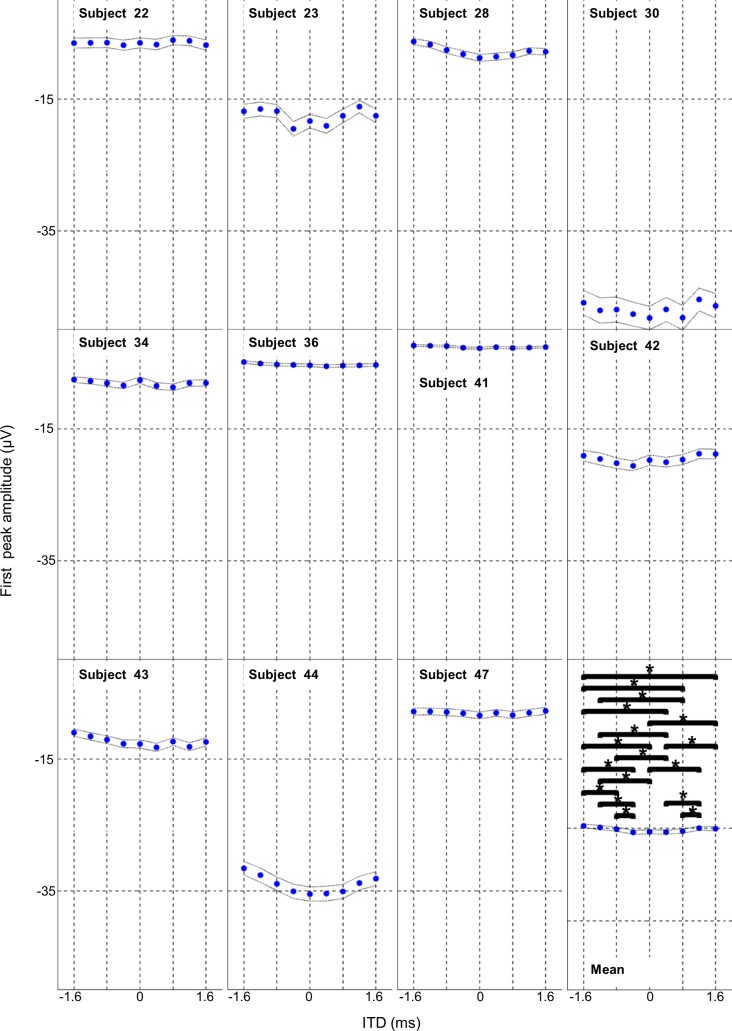
Effect of ITD on average amplitude of first peak of the PAM response. The average baseline to peak amplitude for the 1^st^ peak of the PAM response is shown for 9 ITD conditions for each of the 11 experimental subjects and the overall group mean. Horizontal bars indicate pairwise Tukey’s comparisons and asterisks indicate significance at p<0.05. Fine dotted lines show the 95% confidence intervals.

We also analysed the baseline to peak amplitude for the first (see Fig 1B) and second (see Fig 1C) peak separately. The group data trend seen in the peak to peak amplitude data for higher amplitude responses around -0.4, 0, and +0.4 ITD’s and smaller responses at ITDs greater than _+/- 0.4 ms longer was seen in the first peak amplitude group data but less so in the second peak amplitude group data. The first peak amplitude for the group data was also significantly affected by the ITD condition with ANOVA (F _(10, 8)_ = 13.10, p<0.001). *Post hoc* Tukey tests revealed significant differences between 0, 0.4, -0.4 ms ITDs and +/-1.2 and +/-1.6 ms ITD (p<0.05) for the group data (see Fig 3 lower right Mean panel). Summary data and Tukey p values for pairwise comparison of ITD conditions are shown in the supporting information S1B Table.

**Fig 4:**
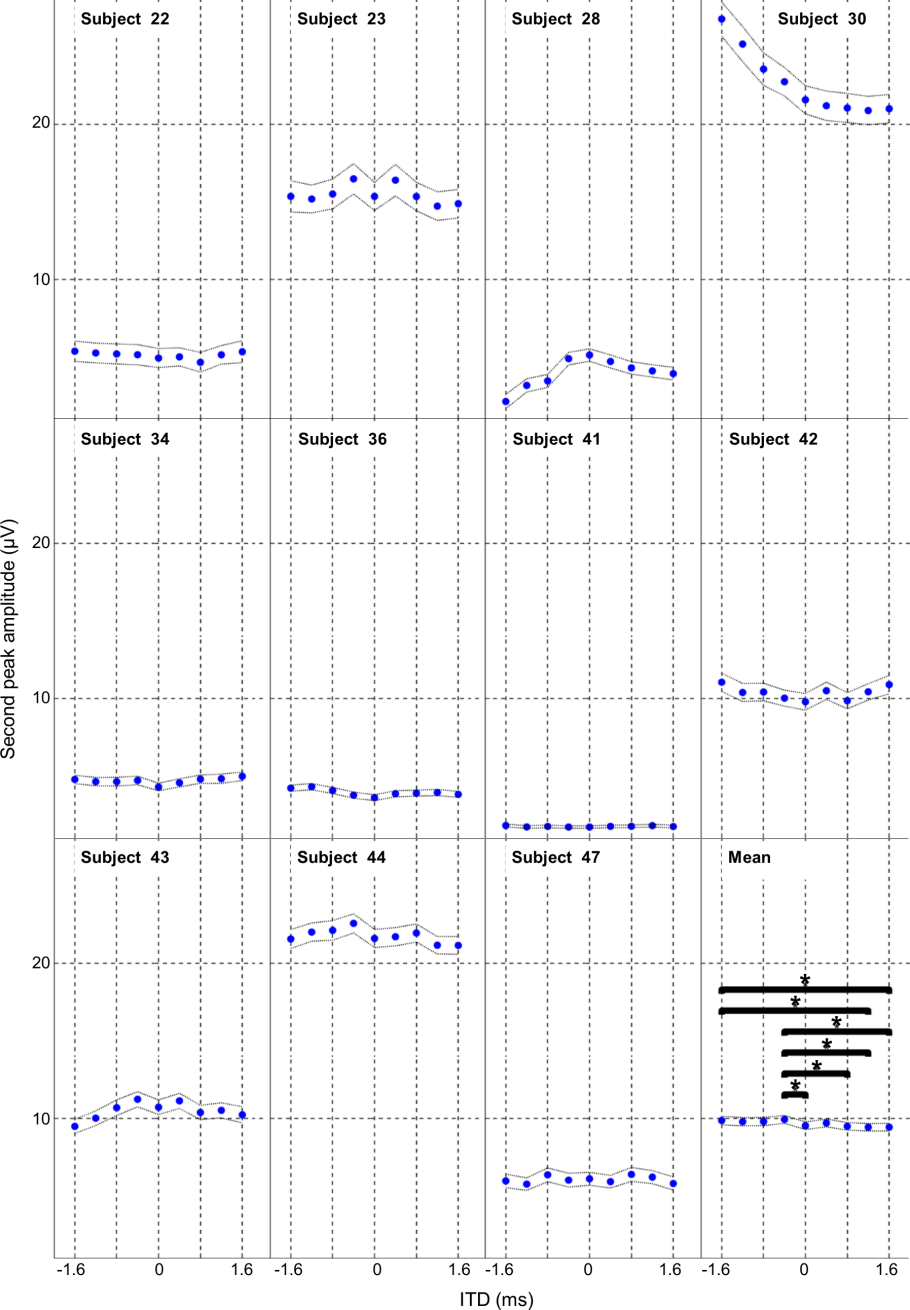
Effect of ITD on average amplitude of the second peak of the PAM response. The average baseline to peak amplitude for the 2^nd^ peak of the PAM response is shown for 9 ITD conditions for each of the 11 experimental subjects and the overall group mean. Horizontal bars indicate pairwise Tukey’s comparisons and asterisks indicate significance at p<0.05. Fine dotted lines show the 95% confidence intervals.

The second peak amplitude for the group data was also significantly affected by the ITD condition (ANOVA, F _(10, 8)_ = 4.65, p<0.001). *Post hoc* Tukey tests revealed significant differences between -0.4 ms ITD and 0.8, 1.2, and 1.6 ms ITD (p<0.05) group data (see Fig 4 lower right Mean panel). The zero ms ITD condition showed significant differences with the -0.4 ms ITD condition only (p<0.05) group data. Summary data and Tukey p values for pairwise comparison of ITD conditions is shown in the supporting information S1C Table.

### First and second peak latencies

**Fig 5:**
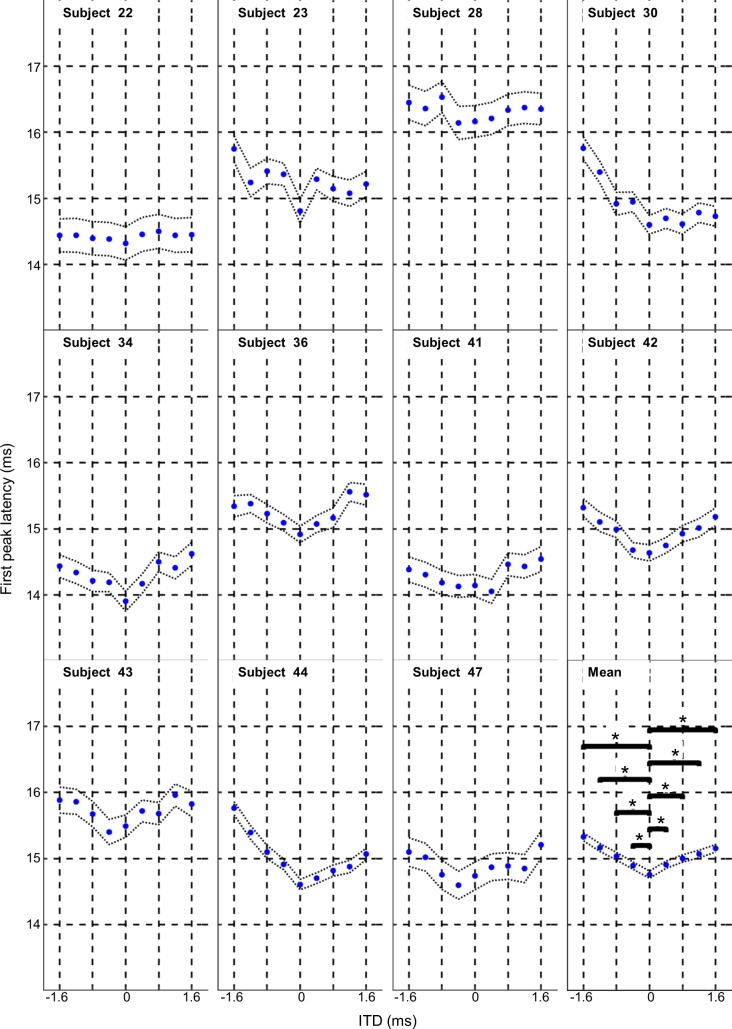
Effect of ITD on the PAM response first peak latency. The average latency of the first peak of the PAM response is shown for 9 ITD conditions for each of the 11 subjects and group mean. Horizontal bars indicate pairwise Tukey’s comparisons (only shows for comparisons between 0 ITD and the other 8 ITD’s conditions) and asterisks indicate significance at p<0.05. Fine dotted lines show the 95% confidence intervals.

**Fig 6:**
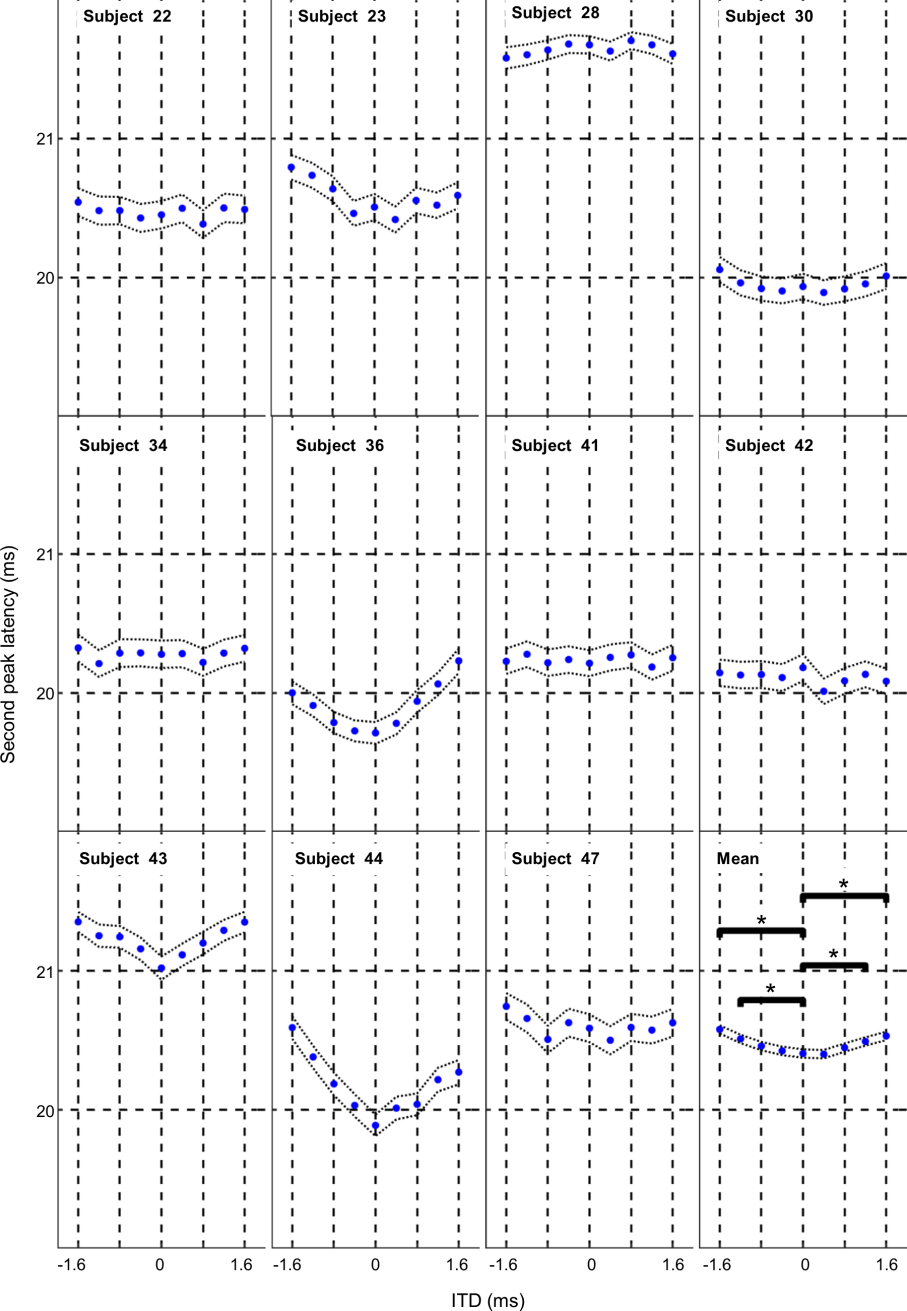
Effect of ITD on the PAM response second peak latency. The average latency of the second peak of the PAM response is shown for the 9 ITD conditions for each of the 11 subjects and group mean. Horizontal bars indicate pairwise Tukey’s comparisons and asterisks indicate significance at p<0.05. Fine dotted lines show the 95% confidence intervals.

In general PAM response latencies for the group data of the first (see Fig 5 lower right Mean panel) and second (see Fig 6 lower right mean panel) peak latencies were shortest for the 0 and 0.4/-0.4 ITD conditions, becoming longer with increasing ITD giving the plots a V-shape centred on 0 ITD. Ipsilaterally delayed stimuli showed a greater latency shift than contralaterally delayed stimuli for the group data.

The first peak latency group data was significantly affected by the ITD condition with ANOVA (F _(10, 8)_ = 23.14, p<0.001). *Post hoc* Tukey tests revealed significant differences between 0 ms ITD and all other ITD’s apart from 0.4ms (p<0.05) for the group data. Summary data and Tukey p values for pairwise comparison of ITD conditions is shown in the supporting information S1D Table.

The second peak latency group data was also significantly affected by the ITD condition with ANOVA (F _(10, 8)_ = 9.90, p<0.001). *Post hoc* Tukey tests revealed significant differences between 0.4 ms ITD and -1.2, and -1.6 ms ITD (p<0.05) for the group data. The 0 ITD condition showed significant differences with the –1.2 and -1.6 ms ITD condition only (p<0.05). The -1.6 ms condition was significantly different from all ITD apart from -1.2 ms. (p<0.05). Summary data and Tukey p values for pairwise comparison of ITD conditions is shown in the supporting information S1E Table.

The group data for first and second peak latencies showed a general trend with the shortest latencies occurring at 0 and 0.4/-0.4 ITD conditions, with longer latencies generated by longer ITD’s giving the plots a V-shape centred on 0 ITD. This V-shape trend was also seen in the individual subject data for the first peak latency (see Fig 5; subject 23, 34, 36, 41, 42, 43, 44, and 47) and second peak latency (see Fig 6; subject 23, 36, 41, 43, 44, and 47).

## Discussion

### Monaural versus binaural PAM responses

As in previous studies, the PAM reflex was evoked by both monaural ipsilateral and contralateral stimulation [36,40,41]. On average contralateral stimulation gave a greater amplitude response than ipsilateral stimulation although this varied between subjects. Insertion of earphones into the ear canal were matched to avoid introducing artefactual intensity differences between the ears. Previous data suggest ipsilateral, contralateral and binaural stimulation showed similar results to ours on response latency but only a small difference between ipsilateral and contralateral stimuli amplitude [36,38]. In terms of function, pinna orienting might be expected mainly to ipsilateral sounds [42], the significant ipsilateral and contralateral activation of the PAM reflex circuitry might suggests a role less to do with spatial orienting and more to with generalised activation as in the startle reflex. This is consistent with the hypothesis that the PAM response is a vestigial orienting reflex that perhaps preserves some startle function in humans [43]. Indeed, the functional role of the PAMR maybe be even more complex than just pinna orienting, or startle and it has been shown to be modulated by eye position and other factors [36,43–46].

Both ipsilateral and contralateral monaural responses are about 0.4 ms quicker than the shortest binaural latency. A very similar binaural delay has been described previously, see Table 1 in [38]. It is unclear if the difference between monaural and binaural delays in the PAM response is due to different physiological integration mechanisms or different circuitry or both. Evidence from the middle-ear muscle reflex pathways show projections from the cochlear nucleus synapse directly or indirectly onto facial and trigeminal motor neurons [47]. However, because the PAM response show sensitivity to ITD it is more likely our data support the theory that binaural activity in the SOC, NLL and or IC drives auditory activity onto PAM motorneurons. More studies are needed to identify the different functional roles of auditory inputs onto PAM motorneurons.

### The PAM reflex was sensitive to ITD

We have shown the biggest PAM amplitude was at zero or +/- 0.4 ms ITDs. This was most apparent for ipsilateral delayed stimuli in the peak to peak amplitude and first peak amplitudes compared to the second peak amplitudes. As ITDs increased above +/-0.4 ms the PAM amplitude decreased. This roll off of PAM amplitude seems to begin at +/-0.4 ms and continue to the maximal ITDs +/-1.6 ms which are beyond the physiological human ITD range. It is possible in our study with more sampling that significant differences might be found between the zero and 0 and +/- 0.4 ms ITD conditions. A previous study examining the role of ITD on the PAM reflex found no effect on the peak to peak amplitude of the PAM response using a +/- 0.4 ms ITD range[48]. This is consistent with our data showing PAM amplitude changes only began at ITD’s greater than +/- 0.4 ms.

Our data is consistent with other studies showing BIC amplitude decreased but continued to be measurable to ITDs greater than the physiological maximum of 0.8 ms [16–18]. Others studies have reported the BIC becomes undetectable between 0.8–1.6 ms [12] and 1.0–1.2 ms [14]. If the PAM response were driven by the same brainstem nuclei that generate the BIC, our data would confirm the studies suggesting binaural temporal processing operates over a ITD range greater that the physiological range of delay introduced by the size of the head, so that it is perhaps more related to the time course of temporal summation of binaural inputs in the brainstem.

We also observed increasing ITD increases the latency of the first and second peaks of the PAM response. The only previous study to investigate the effect of ITD on the PAM response unfortunately did not report latency data. Our data show that the time delay introduced to the PAM response is about between 50–100% of the delay introduced by the ITD. Like previous BIC studies, these data argue against a coincidence detector model with single or double delay lines, which would predict response delays of 50% ITD or 100% ITD respectively [16,18]. Previous data on the ABR peak V and BIC latencies have shown similar trends in both animal and human studies. Modelling studies of animal data further suggest the decrease in amplitude and increase in latency of the BIC can be accurately described by a model assuming inhibitory-excitatory (IE) interactions in lateral superior olive (LSO) [17,18] but not a delay line and coincidence detector model. Modelling BIC data from humans obtained with chirp stimuli instead of clicks gives very similar results to the animal studies[16].

Given the close correspondence in the behaviour of the PAM response to the BIC to varying ITD we suggest it is likely that PAM motor neurons receive functional drive from BIC generating nuclei. Evidence from previous studies make it highly likely that binaural clicks processed through the SOC and NLL drive PAM neuron through an off-shoot of the mainly ascending auditory pathway. Our data do not allow us to conclude if this pathway is relatively direct, lying solely within the pons/medulla, or involves less direct connections routed through the midbrain collicular and/or reticular nucleus previously identified in the pinna reflex circuits.

### Function significance of PAM response to spatial sound

Our results show that activity in the PAM shows sensitivity to ITDs even though pinna movements in humans are vestigial. In our data both contralateral and ipsilateral sound delays generate systematic changes in PAM response latency and amplitude. In cat’s pinna movements are greater for sounds coming from the ipsilateral hemi-field than the contralateral hemi-field. One possible hypothesis which has been put forward posits that the brainstem pathways that evolved to control pinna muscles generate first startle reflexes and then pinna orienting [43]. In humans we might be seeing PAM responses which are generated by brainstem circuits reflecting these two different roles [43].

### Usefulness of the PAM response as a measure of binaural integration

The BIC is one of the only non-invasive, physiological measures of binaural processing that can be used in humans [16] and is useful for different clinical populations [22–24,49–51]. Our data suggest the PAM response sensitivity to ITD is similar to the BIC. As such it may be possible to measure ITD sensitivity in humans using the PAM response instead of the BIC. Although the PAM response is not present is all subjects, when present it has a better signal to noise ratio than the ABR, making it easier to record and reducing recording times as less signal averaging is required [36,52]. The use of chirp stimuli with ITD’s is likely to improve the signal to ratio even further, making the PAM response an alternative method to the BIC for clinical and research studies on human [37].

## Supporting information

S1 Table **Table S1A:** Summary statistics and post hoc p-values for the peak to peak PAM response **Table S1B:** Summary statistics and *post hoc* p-values for the first peak amplitude of the PAM response **Table S1C:** Summary statistics and *post hoc* p-values for the second peak amplitude of the PAM response **Table S1D:** Summary statistics and *post hoc* p-values for the first peak latency of the PAM response **Table S1E:** Summary statistics and *post hoc* p-values for the second peak latency of the PAM response.
(DOCX)

S2 Table Group summary statistics. (XLSX)
