# Supplementary material for: The effect of interaural timing on the posterior auricular muscle reflex in normal adult volunteers
Source: PLoS One. 2018 Apr 4;13(4):e0194965. doi: 10.1371/journal.pone.0194965 (PMC5884533; doi:10.1371/journal.pone.0194965)
Supplement: S1 Table — Table S1A: Summary statistics and post hoc p-values for the peak to peak PAM response Table S1B: Summary statistics and post hoc p-values for the first peak amplitude of the PAM response Table S1C: Summary statistics and post hoc p-values for the second peak amplitude of the PAM response Table S1D: Summary statistics and post hoc p-values for the first peak latency of the PAM response Table S1E: Summary statistics and post hoc p-values for the second peak latency of the PAM response. (DOCX) [file pone.0194965.s001.docx]

Table S1A Summary statistics and post hoc p-values for the peak to peak PAM response

| ITD | Mean | STD | Tukey's comparison P value  Significant p values <0.05 are highlighted in gold | | | | | | | | |
| --- | --- | --- | --- | --- | --- | --- | --- | --- | --- | --- | --- |
|  |  |  | -1.6 | -1.2 | -0.8 | -0.4 | 0 | 0.4 | 0.8 | 1.2 | 1.6 |
| -1.6 | 2.69E-05 | 2.53E-05 |  | 9.99E-01 | 2.12E-01 | **1.85E-06** | 6.04E-02 | **3.03E-04** | 8.61E-02 | 1.00E+00 | 9.97E-01 |
| -1.2 | 2.71E-05 | 2.55E-05 |  |  | 6.27E-01 | **5.78E-05** | 2.97E-01 | **4.89E-03** | 3.73E-01 | 9.99E-01 | 1.00E+00 |
| -0.8 | 2.76E-05 | 2.56E-05 |  |  |  | 8.60E-02 | 1.00E+00 | 6.13E-01 | 1.00E+00 | 2.13E-01 | 7.25E-01 |
| -0.4 | 2.84E-05 | 2.59E-05 |  |  |  |  | 2.75E-01 | 9.85E-01 | 2.12E-01 | **1.85E-06** | **1.18E-04** |
| 0 | 2.77E-05 | 2.56E-05 |  |  |  |  |  | 8.96E-01 | 1.00E+00 | 6.08E-02 | 3.86E-01 |
| 0.4 | 2.81E-05 | 2.57E-05 |  |  |  |  |  |  | 8.40E-01 | 3.07E-04 | 8.40E-01 |
| 0.8 | 2.77E-05 | 2.54E-05 |  |  |  |  |  |  |  | 8.67E-02 | 4.71E-01 |
| 1.2 | 2.69E-05 | 2.44E-05 |  |  |  |  |  |  |  |  | 9.97E-01 |
| 1.6 | 2.71E-05 | 2.50E-05 |  |  |  |  |  |  |  |  |  |

Table S1B Summary statistics and *post hoc* p-values for the first peak amplitude of the PAM response

| ITD | Mean | STD | Tukey's comparison P value  Significant p values <0.05 are highlighted in gold | | | | | | | | |
| --- | --- | --- | --- | --- | --- | --- | --- | --- | --- | --- | --- |
|  |  |  | -1.6 | -1.2 | -0.8 | -0.4 | 0 | 0.4 | 0.8 | 1.2 | 1.6 |
| -1.6 | -1.45E-05 | 1.59E-05 |  | 8.58E-01 | 5.99E-03 | **5.96E-08** | **1.79E-07** | **5.96E-08** | **2.98E-07** | 2.66E-01 | 2.87E-02 |
| -1.2 | -1.49E-05 | 1.63E-05 |  |  | 3.83E-01 | **2.98E-07** | **2.35E-04** | **2.32E-06** | **4.65E-04** | 9.91E-01 | 6.91E-01 |
| -0.8 | -1.53E-05 | 1.63E-05 |  |  |  | 8.54E-03 | 3.64E-01 | 3.96E-02 | 4.63E-01 | 9.33E-01 | 1.00E+00 |
| -0.4 | -1.59E-05 | 1.65E-05 |  |  |  |  | 9.09E-01 | 1.00E+00 | 8.47E-01 | **2.88E-05** | 1.48E-03 |
| 0 | -1.58E-05 | 1.65E-05 |  |  |  |  |  | 9.92E-01 | 1.00E+00 | 1.11E-02 | 1.43E-01 |
| 0.4 | -1.59E-05 | 1.64E-05 |  |  |  |  |  |  | 9.79E-01 | **2.62E-04** | **8.78E-03** |
| 0.8 | -1.57E-05 | 1.65E-05 |  |  |  |  |  |  |  | 1.87E-02 | 2.03E-01 |
| 1.2 | -1.50E-05 | 1.56E-05 |  |  |  |  |  |  |  |  | 9.95E-01 |
| 1.6 | -1.52E-05 | 1.60E-05 |  |  |  |  |  |  |  |  |  |

Table S1C Summary statistics and *post hoc* p-values for the second peak amplitude of the PAM response

| ITD | Mean | STD | Tukey's comparison P value  Significant p values <0.05 are highlighted in gold | | | | | | | | |
| --- | --- | --- | --- | --- | --- | --- | --- | --- | --- | --- | --- |
|  |  |  | -1.6 | -1.2 | -0.8 | -0.4 | 0 | 0.4 | 0.8 | 1.2 | 1.6 |
| -1.6 | 9.86E-06 | 1.04E-05 |  | 9.97E-01 | 1.00E+00 | 9.42E-01 | 5.70E-01 | 9.99E-01 | 1.43E-01 | 5.02E-02 | 6.73E-02 |
| -1.2 | 9.78E-06 | 1.01E-05 |  |  | 1.00E+00 | 4.95E-01 | 9.65E-01 | 1.00E+00 | 6.10E-01 | 3.45E-01 | 4.10E-01 |
| -0.8 | 9.79E-06 | 9.89E-06 |  |  |  | 8.66E-01 | 7.17E-01 | 1.00E+00 | 2.33E-01 | 9.22E-02 | 1.20E-01 |
| -0.4 | 9.93E-06 | 9.66E-06 |  |  |  |  | 3.44E-02 | 5.86E-01 | **2.30E-03** | **4.38E-04** | **6.86E-04** |
| 0 | 9.53E-06 | 9.18E-06 |  |  |  |  |  | 9.36E-01 | 9.98E-01 | 9.68E-01 | 9.82E-01 |
| 0.4 | 9.70E-06 | 9.43E-06 |  |  |  |  |  |  | 5.19E-01 | 2.71E-01 | 3.29E-01 |
| 0.8 | 9.49E-06 | 9.23E-06 |  |  |  |  |  |  |  | 1.00E+00 | 1.00E+00 |
| 1.2 | 9.43E-06 | 9.03E-06 |  |  |  |  |  |  |  |  | 1.00E+00 |
| 1.6 | 9.43E-06 | 9.23E-06 |  |  |  |  |  |  |  |  |  |

Table S1D Summary statistics and *post hoc* p-values for the first peak latency of the PAM response

| ITD | Mean | STD | Tukey's comparison P value  Significant p values <0.05 are highlighted in gold | | | | | | | | |
| --- | --- | --- | --- | --- | --- | --- | --- | --- | --- | --- | --- |
|  |  |  | -1.6 | -1.2 | -0.8 | -0.4 | 0 | 0.4 | 0.8 | 1.2 | 1.6 |
| -1.6 | 15.33 | 2.27 |  | **1.79E-04** | **5.96E-08** | **5.96E-08** | **5.96E-08** | **5.96E-08** | **5.96E-08** | **5.96E-08** | **3.86E-05** |
| -1.2 | 15.17 | 2.26 |  |  | 6.85E-01 | **9.56E-04** | **5.96E-08** | **1.61E-05** | 1.98E-01 | 1.82E-01 | 1.00E+00 |
| -0.8 | 15.04 | 2.20 |  |  |  | 2.90E-01 | **1.37E-06** | **3.18E-02** | 9.97E-01 | 9.97E-01 | 8.65E-01 |
| -0.4 | 14.89 | 2.08 |  |  |  |  | **4.75E-02** | 9.95E-01 | 7.99E-01 | 8.19E-01 | **3.50E-03** |
| 0 | 14.76 | 2.07 |  |  |  |  |  | 3.68E-01 | **7.86E-05** | **9.38E-05** | **5.96E-08** |
| 0.4 | 14.91 | 2.08 |  |  |  |  |  |  | 2.46E-01 | 2.65E-01 | **7.88E-05** |
| 0.8 | 15.00 | 2.09 |  |  |  |  |  |  |  | 1.00E+00 | 3.68E-01 |
| 1.2 | 15.07 | 2.16 |  |  |  |  |  |  |  |  | 3.45E-01 |
| 1.6 | 15.16 | 2.18 |  |  |  |  |  |  |  |  |  |

Table S1E Summary statistics and *post hoc* p-values for the second peak latency of the PAM response

| ITD | Mean | STD | Tukey's comparison P value  Significant p values <0.05 are highlighted in gold | | | | | | | | |
| --- | --- | --- | --- | --- | --- | --- | --- | --- | --- | --- | --- |
|  |  |  | -1.6 | -1.2 | -0.8 | -0.4 | 0 | 0.4 | 0.8 | 1.2 | 1.6 |
| -1.6 | 20.58 | 1.12 |  | 2.75E-01 | **1.14E-03** | **5.84E-06** | **5.96E-08** | **5.96E-08** | **2.38E-07** | **3.00E-05** | **2.59E-04** |
| -1.2 | 20.51 | 1.14 |  |  | 7.29E-01 | 1.09E-01 | **1.05E-03** | **4.41E-05** | **1.16E-02** | 2.28E-01 | 5.00E-01 |
| -0.8 | 20.46 | 1.14 |  |  |  | 9.75E-01 | 2.65E-01 | **4.75E-02** | 6.68E-01 | 9.97E-01 | 1.00E+00 |
| -0.4 | 20.42 | 1.15 |  |  |  |  | 9.19E-01 | 5.26E-01 | 9.98E-01 | 1.00E+00 | 9.98E-01 |
| 0 | 20.40 | 1.15 |  |  |  |  |  | 9.99E-01 | 1.00E+00 | 7.74E-01 | 4.73E-01 |
| 0.4 | 20.40 | 1.14 |  |  |  |  |  |  | 9.32E-01 | 3.18E-01 | 1.20E-01 |
| 0.8 | 20.45 | 1.13 |  |  |  |  |  |  |  | 9.82E-01 | 8.62E-01 |
| 1.2 | 20.49 | 1.13 |  |  |  |  |  |  |  |  | 1.00E+00 |
| 1.6 | 20.53 | 1.13 |  |  |  |  |  |  |  |  |  |
